# Metallic Dies

**Published:** 1855-07

**Authors:** P. H. Austen


### ARTICLE III.

*Metallic Dies. An Essay read before the Society of Associated Alumni of Dental Colleges, March 1st,* 1855. By Prof. P. H. AUSTEN.

GENTLEMEN—Appointed, at your last meeting, to prepare an essay upon some practical subject, I have chosen one which has not, as seems to me, received from the profession generally, a sufficiently careful consideration. The selection of metals suitable for metallic dies, and the precautions necessary to insure their perfect accuracy.

I shall not attempt to exhaust the subject, for it is too comprehensive: nor presume to claim for myself infallibility, in every assertion that I may venture to make. In warning you against errors in practice and mistakes in theory, I desire to exercise towards others the courtesy due from every one to a professional brother; nor would I seek, by indulging in presuming arrogance or offensive personality, to add another to the list of those who, calling themselves friends of truth, become, her worst foes.

This Society is made up, almost exclusively, of those who are yet young in professional experience. Myself not a Mentor in years, may I be pardoned if I here urge briefly upon you modesty in the assertion of your own views however correct, and a gentlemanly forbearance in rectifying the errors of others. Few things are more repulsive than a young man's conceited assumption of superiority in the midst of his grey-haired seniors. If some veterans in dental practice are far behind the newly fledged graduate, in a knowledge of the capabilities of his art and its more approved appliances, this only proves the advanced condition of the science, not any necessary inferiority of individual talent. Any tyro of fair capacity may, in a few years, master all that it took NEWTON a life to learn, and even more than this; yet, among the many would-be Newtons of the age, there are none whose fame will dim the lustre, that brightens the memory of the unpretending philosopher of old. Let us learn humility, standing with him on the shore of the unexplored ocean of science, where at best, "each generation may pick but a few pebbles." Rest assured, gentlemen, these "pebbles" are never put to a worse use, than when you are engaged in throwing them at one another's heads. Craving your indulgence for what may seem a somewhat irrelevant introduction, I proceed at once to define some of the terms used in this essay.

The term IMPRESSION is applied only to the copy taken directly from the mouth, whether in wax, plaster, or gutta percha. The scope of this dissertation will not allow any description of this process. Suffice it to say, that inaccuracy here must necessarily vitiate the result, however correct the subsequent steps. Too often is it the case that the cause of failure, properly lying here, is unjustly attributed to the shrinkage of the metallic dies, the operator blaming the innocent metal, sooner than admit the possibility of *his* taking an inaccurate impression.

The term CAST is applied only to the plaster cast, which is taken from the impression, and which is, or should be, a facsimile of the natural organs. In no case is the word used in the sense of "casting" and applied to metals. The terms "metallic casts, zinc casts," &c. are carefully avoided, in order that this word may not be associated with any two distinct stages in the regular process of getting up a plate.

The terms MOULD or MATRIX are applied to the cavity into which the metal is poured to form the die. This cavity which may be made in sand or plaster, or a mixture of both, is formed by the cast, and the process is termed MOULDING. These words are certainly preferable to the circumlocutions in common use "taking the impression in sand," &c. necessary, where the word impression is used, to avoid confusion.

The terms DIE and COUNTER-DIE are applied to the metallic masses between which the plate is swaged: the first, a fac-simile of the *cast,* and by consequence of the natural organs; the second, its reverse, and a fac-simile of the *impression.* The terms "male cast, female cast," apart from the double use of the word "cast" are objectionable, on the score of good taste, as very inelegant: whilst the terms "zinc cast, and lead cast," fail to be significant of the difference between the two dies, whenever zinc is no longer used for the one, or lead for the reverse. That part of the die brought into contact with the plate to be swaged is technically called its FACE or "front:" the opposite side, called with convenient brevity the "back," in the die, receives the blow of the swaging hammer, and in the counter-die rests upon the block or anvil.

This attempt to define the meaning of a few words in constant use, is here offered, not simply as preparatory to the remainder of this essay; but for your general adoption, as calculated to do away, in some degree, with that vague, and confused use of terms so frequently met with. A language is rich in so far as it has a word for every shade of thought, and energetic in proportion as it expresses fully an idea without circumlocution. It is these two qualities that give to the Greek language its superiority, and which are possessed by our own tongue in a marked degree. What is true of language in general, may be said of so much thereof as art and science employ to express their technicalities. Each object or process should have its distinctive term, nor should two words be used where one will suffice to express the idea.

We have to deal at present with the class of so called "baser" metals, whereby, in the form of dies, we mould the "nobler" ones to our uses. Touching the propriety of this time honored classification, it need only be said, that the world is not a whit more unjust to metals than to men. Few words are suggestive of more baseness than the noble metal gold, and in the civilization of the world no metal has done a nobler part than the base one, iron. Neither is "nature's nobleman" always or only found among those whom social or political distinction calls noble.

From this class of baser metals must be selected those which best answer the requirements of the dentist. Fusibility is one important requisite, inasmuch as the practitioner can rarely command the intense heat necessary for the fusion of sufficient quantities of unalloyed copper or iron. Striking from the list all those infusible at a red-heat, there are eleven remaining. Of these sodium and potassium are inadmissible, because of the impossibility of preserving their metallic form on exposure to the air: mercury, because of its liquid condition: arsenic, because it will volatilize before reaching the fusing point: and Tellurium, because of its variety and consequently costliness, without possessing any compensating advantages. There remain only six, viz.

| | |
|---|---|
| Tin, | Lead, |
| Cadmium, | Zinc, |
| Bizmuth, | Antimony; |

to which may perhaps be added copper and iron, as having a very limited use in the composition of certain alloys. Before entering into the relative merits of these six or eight metals, it will be well to inquire more at length into the properties required in metallic dies.

The first essential requisite of a die,* is that it shall possess

* Let it be noticed that this word, wherever used without the prefix, "metallic," designates the die proper, as distinguished from the counter-die.

sufficient HARDNESS to force the plate into perfect adaptation, without any material change in its own form. Did the plate come nearly into contact with the whole face of the die, from the first, its yielding under compression would be of less moment, as all parts would yield alike. But such is far from being the actual condition, and the first blows impress their whole force upon the prominent parts of the die. The more marked the irregularities of the mouth, the more essential does this requisite become, especially since the resistance of the plate is increased in proportion to the number and depth of its corrugations. The kind of hardness, in respect of which the different metals and their alloys are to be compared, is not so much the hardness that resists the action of a file or other abrading tool, as that which resists compression under repeated blows. And, as these are not necessarily identical, hence the importance of a classification different from that found in our books, where the metals are graded in the same manner as minerals.

To those of you who may desire to benefit yourselves and your profession, by a series of experiments upon this point, allow me to suggest briefly the simple apparatus necessary. First, a small ingot mould to secure uniformity of size and shape in the pieces of metal experimented upon. Second, a steel pointed punch with a circular face, $\frac{1}{8}$ or $\frac{1}{4}$ inch in diameter, fixed over a firm metallic base upon which the ingots rest, and capable of moderate vertical motion. Third, a 5 lbs. hammer, arranged either in an upright slide, or on the end of a movable arm, so as to fall from a given height upon the head of the punch. Lastly, a micrometer screw, capable of measuring thousands of an inch, with which to determine the depth of the indentation made by the punch. The hardness of any metal varies inversely as the depth of the indentation. With this simple rule, a scale may be readily formed. Conjecture is thus replaced by certainty—a substitution of utmost importance in any department of science.

A point which demands notice here, is the proper relative hardness of the die and the counter-die. The same metal is often used for both dies and, in some instances, the harder metal for the counter-die. A little reflection will show that there should always be a decided difference in favor of the die. The faces of the two dies before swaging, are in contact at all points. But, separate the two in a vertical direction, and this uniformity is no longer preserved. The horizontal portions corresponding with the floor of the palate and the top of the alveolar ridge will be most widely separated; the sloping palatine surfaces less so, in proportion to their abruptness, and the outer vertical sides of the alveolus, not at all. Now, as the plate to be swaged has a very appreciable thickness, one or the other die must yield in those parts which approach too closely, before the dies can be brought together again with a uniform thickness of plate between. Of course all such partial yielding is, just so far, a distortion, and if it takes place in the die, which it will be remembered is the fac-simile of the mouth, it must of necessity affect the accuracy of the plate; hence, to avoid this danger, the die should be decidedly the harder of the two, that the greater part if not all of the compression may take place in the softer counter-die.

An argument urged in favor of using a metal harder than lead or tin for the counter-die, is, that it lessens the liability of the die to "rock" under the force of an occasional one-sided stroke of the swaging hammer. The operator who does not know how to avoid ever making such a stroke, has a simple lesson in dental mechanics yet to learn. It is this same unskillfulness that causes many to bury one die too deeply into the other—a practice fraught with much annoyance, without a single redeeming advantage.

The second requisite in a metallic die, is FUSIBILITY at a moderate temperature. As before remarked, comparatively few practitioners can have ready access to means sufficient to fuse metals (in 5 pound masses) more refractory than zinc. And there are hundreds whose labors would be materially lightened, if furnished with an alloy equally hard as zinc, which would yield to the heat of the spirit lamp. The city dentist, surrounded with every appliance, and having at his command the means of making cast iron dies if necessary, often fails to appreciate the slender opportunities of his village brother, whose movable laboratory bears to his own some such relation as a medicine chest does to an apothecarium. To relieve the embarrassing difficulties of the one, is a greater boon to the profession than to add to the ingenious contrivances of the other.

But a second argument in favor of metals, that fuse at a low temperature, is the lessened amount of shrinkage. The lower the melting point, *ceteris paribus*, the less a die will shrink. All metals, below their point of fusion, are subject to the general law of expansion by heat and contraction by cold. True, the rate of expansion varies, but with every allowance for such variation, the metal that "sets" at 770°, and has to lose 700 degrees of heat to reach the mean temperature of the air, will shrink much more than one that cools through only 200 degrees. What shall we then say of copper, shrinking through 1900 degrees, and brass through 1800; will their increase of hardness compensate for their great contraction? We think not.

As regards the relative fusibility of the two dies, it is much safer that the one last poured should be the more fusible; though, with care, metals of equal fusibility may be used, or the one last made might be a few degrees less fusible than the first. In any such case, the first die should be cool, and carefully coated on its face with whiting or lamp smoke. There is not, however, we think, any necessity for incurring such risk. Some operators, by the dipping process, or otherwise make their counter-die first; others again, by the process of moulding, first cast the die. Either may, by reference to the table subsequently to be given, select two metals or alloys, such that, whilst in both cases the counter-die shall be the softer, in each the metal first used shall be the more infusible.

The third important requisite in a metallic die is non-contraction upon cooling so far as this is attainable. It is the yielding softness of the mucous membrane that permits the work of the most skillful to be retained firmly in place. It is this same accommodating quality that also favors the unskillful, whereby the mouth in time adjusts itself to a piece, which has not in the first place been properly adjusted to it. The extent of this adaptability is well shown in the fact, that artificial dentures made entirely of porcelain, have sometimes been worn, notwithstanding the distortion which the furnace necessarily produces, and for which it is quite impossible to make accurate allowance. Now, since no die, except steel, which is, of course, impracticable, possesses a degree of hardness resisting all compression under the hammer; since also none is free from some contraction upon cooling, it is evident that were the surface of the mouth as hard as metal, it would be absolutely impossible to bring a plate into contact at all points by the process of swaging. It is your duty not to abuse that facility of adaptation; nor should you ever rest satisfied with work that is barely able to retain itself in the mouth.

I may seem to many, in the three "requisites" above enumerated, to indulge in unnecessary refinement. One will say he has never used any other metals than zinc and lead, and he has never found the shrinkage of the zinc a source of inaccuracy. Another has always used his two dies of the same metal, and yet his plates always fit. A third, who never uses any material harder than tin, pewter, or type metal, is uniformly successful. One of three inferences from such statements must be true; either the operators have a limited experience; or they fail to distinguish between a firm and unstable adaptation; or they are deficient in candor. The profession abounds in men who speak thus confidently of their own particular methods. Gather them in convention and let each speak of the merits of his own plan, and you might well think that dental science had reached its *ultima thule* of perfection: but hear them comment each upon the plans of the rest, and you then might wonder if there had really been any progress made since the days of Desirabode. "This remedy never fails to restore the most broken constitution, in a very short time, to a condition of perfect, healthful vigor." Such is the language of the charlatan, who would have the world think that one panacea will suffice for all the ills that flesh is heir to. Such, virtually, is the spirit of every one who assumes for any process a too universal application, and who, by denying the necessity for improvement, would check the progress of scientific discovery.

Now there are many cases in which moderate shrinkage will not appreciably affect the accuracy of adaptation. Contraction takes place uniformly throughout the whole mass, and its amount between any two points is proportioned to the distance. The greatest distance practically met with is between the extremities of a lower plate, and consequently the most contraction. Hence the frequency with which in such cases, especially where the investing membrane is hard, the hazardous, uncertain method of bending with the fingers is resorted to. But in an upper plate, where the arch is not too deep or wide, and the mucous membrane not too firm, the softness of this membrane will adapt it to any inaccuracy of the plate from under a zinc die, whether caused by contraction or compression. Unfortunately for the practitioner of one idea, there are some very deeply arched mouths, some with the mucous membrane nearly as hard as bone, and some in which there is an annoying combination of hardness in one part with softness in another.

It has been suggested among the remedies for this source of inaccuracy, that the thickness of the varnish on the cast may be made to compensate. A moment's reflection must show the error of such a calculation. On the outside of the alveolar ridge this compensation might take place, but on the inner sides of the opposite alveoli, the varnish unites with the shrinkage, to lessen the space between them, whilst the distance between the plane of the ridge and the floor of the palate remains the same as in the unvarnished cast. The same remarks will apply equally to another suggestion, namely, where sand is used, "to so loosen the cast as slightly to enlarge the mould." Now, though varnish as a corrective of shrinkage is worthless, yet, when properly applied, it does no harm, except when the counter-die is taken at once from the cast. But this latter suggestion is more than worthless, it is very reprehensible. This is an important point and worthy of more than passing notice.

Every motion communicated to the cast in the act of moulding, beyond the slight jarring sufficient to detach the sand from its face, impairs the accuracy of the die. The enlargement is never uniform, but is greater around the ridge, thus practically deepening the arch and giving to the plate that "rocking" motion, so frequent a source of annoyance. It is practiced by many, that the cast may be readily withdrawn. But if moulding in sand involves any such necessity, 'twere much better to return to the older practice, which takes the die at once from the cast. The custom of some dentists to trust their casting to the hands of a brass founder, is reprehensible, not only because of the great contraction of brass or copper, where these metals are used, but also because such men scarcely ever can be induced to take that care, the necessity for which they cannot appreciate, and which indeed, in their every day work, would be wholly uncalled for. Every cast should be so shaped as to part freely from its mould, without the least appreciable motion being given in the act of loosening. Peculiar conformations of the alveolus must be met by methods, which it would be irrelevant here to describe, but no difficulties of shape can justify the enlargement of one part of the mould in order to withdraw the cast from another. Of those whose habit it is to remove the cast from the sand between the thumb and forefinger, it is sufficient to say we trust their sin is one of ignorance and not of choice.

One other popular error, relative to the contraction of the die, demands notice. It is said the metal must not be poured too hot into the mould, least there be an unnecessary shrinkage. True, the metal should not be too hot, but for a different reason, to wit: the danger of spoiling the die by the too rapid evolution of vapor where the sand is used, without any previous drying. So far as accuracy in other respects is concerned, the hot poured metal will give the most correct die, because by reason of its greater fluidity, more searching. This is evident upon comparing the smooth surface of a cold poured die with the granulated surface of one poured hot, in which the minutest grain has left its impression. As regards shrinkage, there is not, as respects the *face* of the die, the slightest difference, though on the back, it is very perceptible in the depth of the central depression. What are the changes that take place during the cooling of the metal?

Ice melts at 32° Farenheit, and water solidifies at the same temperature. So of all metals it may be said their points of solidification and of liquefaction are identical. Selecting zinc for illustration—it cannot be poured at a lower temperature than 773°, nor at however higher degree poured, can it solidify until it reaches that point. Now, in every liquid mass, contraction by cold is shown by subsidence of the fluid, not by any change of relation to the lower parts of the receiving vessel. The mercury in the thermometer bulb will constantly fill it, whether at zero or boiling point, but any decrease of temperature below its freezing point, will cause the mercury to become smaller than the interior of the bulb. The molten zinc will be in uniform contact with every part of its matrix, and will be level on its upper part until solidification takes place, beginning at the outer surfaces in contact with the sand and the air. The die then assumes its fixed relation, and at 773° is an exact duplicate of the cast, supposing the process of moulding to have been correctly performed. But as the concentric layers cool, they continue, being yet fluid, to subside, leaving that depression characteristic of the zinc die, and which is deepest at the point last solidifying. The heat conducting power of the metal keeps it at very nearly the same temperature, till it has become solid throughout; after which the change upon the face of the die begins, and the whole mass becomes smaller by contracting upon itself through 700°, till it reaches the average temperature of the laboratory. The shape of the back of the die may vary, as the upper layer of the metal cools more or less rapidly. If suddenly chilled, a level will cover the central depression, or the refuse of the ladle may hide it. A more gradual cooling gives a dished appearance; but if by covering the casting instantly with a large piece of ignited charcoal, this surface-cooling is altogether prevented; it will then be perfectly level. Whether the back of the die be level or concave, or with an abrupt depression in its center, is a matter of no moment to one who practices the proper method of swaging. The counter-die of lead or tin presents no such irregularities on the back, because, cooling less gradually, the upper surface subsides together, and the whole mass solidifies almost instantly. The same difference is observable in melting as in solidifying; zinc melting gradually, and lead more suddenly.

In the selection then of a metal or alloy for the die, with a view to secure the least degree of shrinkage, regard must be had first to the rate of contraction; secondly, to the melting point. There is no need to search for an alloy, which like ice, expands in the act of solidifying, as antimony is supposed to do, and in a less degree, type metal. Of this last named alloy, it is almost universally said that it fills the sharp outlines of the delicate matrix of the type founder, by virtue of its expansive property. But some recent researches of Mr. Charles Tomlinson, satisfactorily show that the addition of the antimony does not render the contractile lead expansive, but only lessens its rate of contraction. More than this we cannot hope to secure by any addition of antimony to lead, tin or zinc. But, both for type founder and the dentist, this is quite sufficient. The sharply defined face of the type is brought out by a peculiar upward jerk of the matrix, at the instant of pouring, and the antimony is useful in lessening the tendency of lead to cool suddenly, thus allowing time for it to search the finest lines of the mould. This last property of the antimony, next to the increased hardness it imparts, is most useful to the type founder; its contraction upon cooling, being uniform in all types would give no trouble, if in moderate degree. What is done in the foundry by the quick upward motion of the hand, is accomplished in the dentist's laboratory by the mere weight of the metal. The small quantity of metal, in the first case, renders some such expedient necessary; but the molten metal of the die, in the first place, being in so much larger mass, retains its fluidity long enough to search every irregularity of the mould; in the second place, its face is kept in close contact with the matrix, whilst passing from the liquid to the solid state, by the pressure of the three or four pounds of superincumbent metal; lastly, as has been previously shown, all contraction prior to solidification, affects the back of the die and not its face.

A fourth and last property, which will be briefly noticed as requisite in a metallic die is COHESION. Metals deficient in this are brittle and will not stand the number and force of blows sufficient to swage a gold or silver plate. Many dies, however, possessing a sufficient cohesive force, will not stand the sledge hammer violence, which some operators think it necessary to employ. This undue violence, together with a faulty shape of the die itself, cause many to class zinc among those metals too brittle for use; whereas a skillful manipulator has no occasion even to break a properly shaped zinc die. Antimony and bismuth are alone unfit for use, and if added in too large proportion, will render alloys too brittle. This effect must be carefully remembered in our assays to find among the alloys one which to greatest hardness shall unite fusibility and least contractility; for these valuable properties are of course worthless if its brittleness prevents their being made available.

We pass now to the investigation of the physical properties of each of the six metals—tin, cadmium, bismuth, lead, zinc and antimony, attempting to assign to each its relative value, and its proper application in the dental laboratory. Inseparable from such an investigation, is the subject of metallic alloys, generally of much more value than the pure metals themselves, and well worth a series of careful and accurate experiments.

[To be continued.]

