# Erratum

**Published:** 1856-07

**Authors:** 


* Erratum.—In July No., 1855, page 372, 24th line, for "variety and consequently costliness," read scarcity and consequent costliness. This conclusion of our article having been greatly altered from the original report, is worded in terms more suited to the readers of the Journal.

